# Predation has small, short-term, and in certain conditions random effects on the evolution of aging

**DOI:** 10.1186/s12862-021-01815-8

**Published:** 2021-05-17

**Authors:** Peter Lenart, Julie Bienertová-Vašků, Luděk Berec

**Affiliations:** 1 Research Centre for Toxic Compounds in the Environment, Faculty of Science, Masaryk University, Kamenice 5, Building A29, 62500 Brno, Czech Republic; 2 Department of Experimental Biology, Faculty of Science, Masaryk University, Kamenice 5, 62500 Brno, Czech Republic; 3 Centre for Mathematical Biology, Institute of Mathematics, Faculty of Science, University of South Bohemia, Branišovská 1760, 37005 Ceske Budejovice, Czech Republic; 4 Department of Ecology, Biology Centre, Institute of Entomology, Czech Academy of Sciences, Branišovská 31, 37005 Ceske Budejovice, Czech Republic

**Keywords:** Predation, Aging, Trade-off, Reproduction, Fecundity, William’s hypothesis, Antagonistic pleiotropy

## Abstract

**Background:**

The pace of aging varies considerably in nature. The best-known explanation of the evolution of specific rates of aging is the Williams’ hypothesis suggesting that the aging rate should correlate with the level of extrinsic mortality. However, the current evidence is inconclusive with various examples where the Williams' hypothesis seems to be correct and where it doesn’t. Here we explore the relationship between extrinsic mortality and aging rate by developing a simulation model of the evolution of aging rate in prey subject to predation.

**Results:**

Our results suggest that more intense predation leads to the evolution of faster pace of aging in prey. However, this effect slowly vanishes when the predator diet breadth is allowed to evolve, too. Furthermore, in our model, the evolution of a specific aging rate is driven mainly by a single parameter, the strength of a trade-off between aging and fecundity. Indeed, in the absence of this trade-off the evolutionary impacts of predation on the prey aging rate appear random.

**Conclusions:**

We show that the William’s hypothesis appears valid when there is a trade-off between aging and fecundity and predators and prey do not coevolve. However, we also show that when the prey and predators coevolve or if there is no trade-off between aging and fecundity the William`s hypothesis is no longer applicable.

**Supplementary Information:**

The online version contains supplementary material available at 10.1186/s12862-021-01815-8.

## Background

Aging, defined as an age-dependent increase in the mortality rate [1], is a widespread biological phenomenon. However, it seems that some species do not age at all [2–5] or at least do not age at the scale comparable with others. In addition, in species that do age, the actual pace of aging is quite diverse [2, 6], which suggests that the aging rate may be a trait malleable by evolutionary forces. The field focusing on the evolution of aging is deeply divided into two seemingly irreconcilable groups [7], one suggesting that aging is adaptive [8–15] and the other (the mainstream one) that it is not [16–22]. Nevertheless, both groups agree that a better understanding of the mechanisms driving the evolution of the pace of aging would certainly provide an important insight into how aging operates and how it can be modulated.

The best-known prediction concerning the evolution of specific rates of aging is the Williams’ hypothesis stating that “low adult death rates should be associated with low rates of senescence, and high adult death rates with high rates of senescence” [17]. While there are studies providing evidence in support of this hypothesis [23–26] there are also works showing it is situation-dependent [27, 28], results putting it in doubt [29], famous contradictory examples [30], an article stating it is entirely wrong [31] and works criticizing that article as an unjust oversimplification [32, 33]. In other words, the discussion about the validity of Williams’ hypothesis and the role of extrinsic mortality in the evolution of aging is still not settled.

In this article, we develop a novel mathematical model that simulates the evolution of the aging rate under various ecological scenarios. In particular, we ask how the presence of predators affects the aging rate in their prey, and how this may in turn shape predators’ consumption preferences for prey of different age. Also, we ask how these relationships are modulated by commonly assumed life history trade-offs, an aging rate-reproduction trade-off in prey and a searching effort-maintenance trade-off in predators. In other words, we explore the validity of Williams’ hypothesis under specific ecological conditions.

## Methods

To address our questions, we develop an agent-based simulation model of a predator–prey interaction that allows all prey and predator individuals and hence their phenotypes to be modelled explicitly and their competitive, predatory and phenotypic dynamics to be followed over time. Time is discrete, with the time step corresponding to the age increment of 1 and with all relevant rates and probabilities defined on the per time step basis. We consider populations composed of $N$ prey and $P$ predators. Although we do not account for males and females, we require that in both species two individuals need to meet and mate to reproduce and formally thus consider simultaneous hermaphrodites. All model parameters and variables are summarized in Table 1.

**Table 1:** Default parameter settings used in our simulation model

Parameter	Meaning	Value(s)
$N$	Fixed prey population size	1000
$P$	Fixed predator population size	Varies
${d}_{0}$	Per time step probability of dying at age 1 for prey individuals	0.01
${k}_{d}$	Rate at which mortality of prey individuals increases with age	Trait
${b}_{0}$	Fecundity of prey individuals at age 1	1
${p}_{m}$	Probability of mutation upon offspring production	0.5
${\sigma }_{m}$	Variance in trait upon mutation	0.01
${w}_{d0}$	Minimum predator attack rate	0.001
${w}_{1}$	Maximum predator attack rate	0.01
${k}_{w}$	Flexibility in the predator attack rate	Trait
$m$	Per time step probability of dying for predator individuals	0.02
${e}_{0}$	Assimilated energy from prey of age 1	1
$x$	Trade-off strength in prey trait ${k}_{d}$	0, 0.001, 0.01
$y$	Trade-off strength in prey trait ${k}_{w}$	2
${e}_{1}$	Energy needed to produce one predator offspring	1
${k}_{e}$	Marginal rate in assimilated energy with prey age	0.1
$k$	Maintenance parameter in predators	Varies

### Prey demography

Prey individuals are characterized by age $a$, and their phenotypes are assumed to differ according to the mortality rate profile. We define the probability that a prey individual of age $a$ dies during a single time step from intrinsic reasons as

1 $$d\left( a \right) = d_{0} + \left( {1 - d_{0} } \right)\frac{{\left( {a - 1} \right)}}{{\left( {a - 1} \right) + k_{d} }}$$

**Fig. 1:**
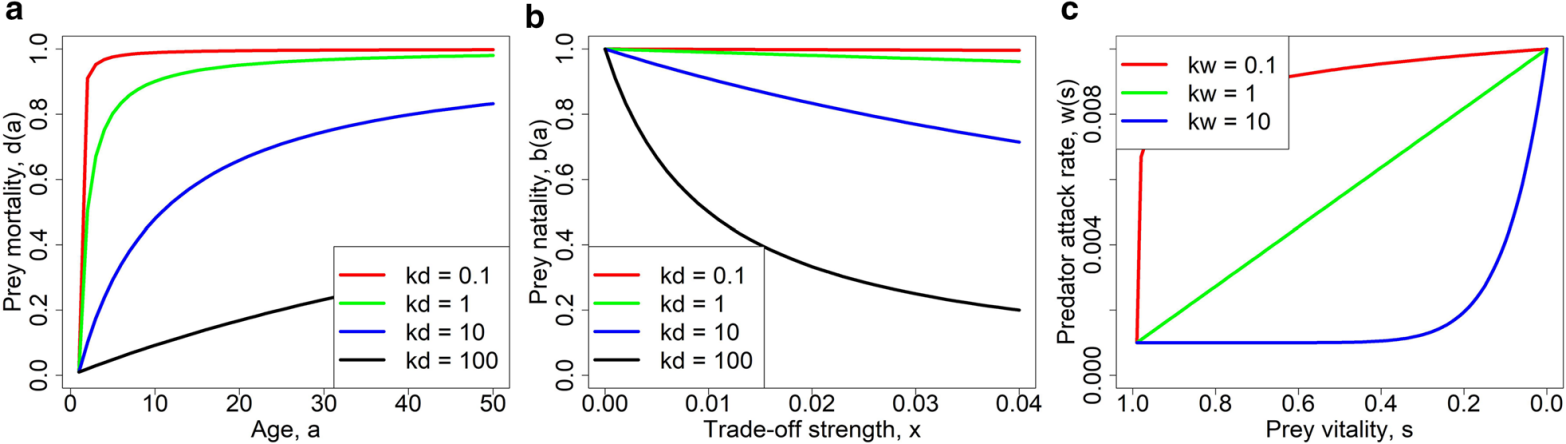
Functional relationships describing various model elements. **a** Intrinsic prey mortality described by Eq. (1), **b** prey fecundity described by Eq. (2), and panel **c** plots predator attack rate of prey described by Eq. (3). Parameters: ${b}_{0}=1$, ${d}_{0}=0.01$, ${w}_{d0}=0.001$, ${w}_{1}=0.01$. We note that prey vitality decreases with age as $s(a)=1-d\left(a\right)$

This function increases in a decelerating way from $0<{d}_{0}<1$ for $a=1$ to $1$ as $a$ grows large (Fig. 1a). Different prey phenotypes are distinguished by different (positive) values of the parameter ${k}_{d}$ which determines the rate at which mortality increases with age. Specifically, higher ${k}_{d}$ means less steep slope at $a=1$ and slower approach of the limiting value $1$, and hence slower aging (Fig. 1a). Thus, the evolution of aging rate is in this setting equivalent to the evolution of parameter ${k}_{d}$ within the prey population.

Since slower aging need not come for free yet rather have a negative impact on another prey trait, we assume that an increase in the aging rate parameter ${k}_{d}$ (i.e. slower aging) implies lower individual fecundity and vice versa. Many evolutionary biologists commonly assume a trade-off between reproduction and aging as it is suggested by both antagonistic pleiotropy [17] and the disposable soma theory [18, 19]. Nevertheless, there is a controversy about whether such a trade-off [34–36] appears in nature. Thus, we explore our model with as well as without this specific trade-off. There are many alternative ways of how to model this relationship. Denoting by $x$ the strength of trade-off between the aging rate and fecundity, we use the following relationship (Fig. 1b):

2 $$b\left( {x,k_{d} } \right) = \frac{{b_{0} }}{{1 + x k_{d} }}$$

We further assume that offspring mortality in their first age (i.e. time) step is included in the parameter ${b}_{0}$ and Eqs. (1) and (2) are thus applicable to prey individuals of age $a\ge 1$.

### Predation and predator demography

Predator population is assumed unstructured with respect to age but the attack rate of predators towards prey depends on the age of prey $a$. Specifically, ‘older’ prey individuals, considered weaker, are assumed to have reduced abilities to escape predators. But ‘older’ has a relative meaning, as various prey individuals may age at different rates, so we should rather say that less vital individuals are assumed to have reduced abilities to escape predators. Such a distinction between chronological age and physical performance is well supported by existing data: a recent study performed on a sample of 126,356 subjects showed that age estimated based on exercise stress testing performance was a better predictor of mortality than chronological age [37]. We thus consider the prey survival probability at age $a$, $s(a)=1-d\left(a\right)$ as a proxy of prey vitality at that age. Note that different prey phenotypes (i.e. prey with different values of ${k}_{d}$) may have the same vitality at different ages which is exactly what we aim here for. We model the predator attack rate of prey as a decreasing function of the prey vitality $s\left(a\right)$:

3 $$w\left( {s\left( a \right)} \right) = w_{d0} + \left( {w_{1} - w_{d0} } \right) \left( {1 - \frac{s\left( a \right)}{{1 - d_{0} }}} \right)^{{k_{w} }}$$

Here, ${w}_{d0}$ and ${w}_{1}$ are minimum and maximum predator attack rates, while the parameter ${k}_{w}$ allows for flexibility in the predator attack rate with respect to the prey vitality; ${k}_{w}=1$ corresponds to a linear function, whereas ${0<k}_{w}<1$ and ${k}_{w}>1$ correspond to concave and convex forms of $w(s(a))$, respectively (Fig. 1c). Individual predators may differ in the parameter ${k}_{w}$, thus representing diet breadth. However, all predators have at least a small preference for the older, less vital prey. The parameter ${k}_{w}$ thus determines predator phenotype, and we are interested in its evolution, too.

With this, the probability that a prey individual $j$ escapes predation within a time step is

4 $${\text{exp}}\left( { - \mathop \sum \limits_{i} w_{i} \left( {s\left( {a\left( j \right)} \right)} \right)} \right),$$

where the index $i$ runs over all predators. The probability that when a prey individual $j$ is consumed it is a predator $k$ that consumes it, is

5 $$\frac{{w_{k} \left( {s\left( {a\left( j \right)} \right)} \right)}}{{\mathop \sum \nolimits_{i} w_{i} \left( {s\left( {a\left( j \right)} \right)} \right)}}$$

Once all prey individuals are tested for being consumed or not, the mean number of offspring a predator produces is calculated as proportional to the total energy obtained from prey consumption:

6 $$f = \frac{{\left( {1 - k} \right)\mathop \sum \nolimits_{i = 1}^{C} e\left( {a_{i} } \right)}}{{e_{1} }}$$

Here $C$ is the number of prey individuals a predator consumes in a time step, $e({a}_{i})$ is an energy assimilated from the *i-*th consumed prey of age ${a}_{i}$, ${e}_{1}$ is an energy needed to produce one predator offspring, and $k$ is a proportion of the energy assimilated from food that is used for predator maintenance rather than reproduction. We assume for simplicity that $e\left(a\right)={e}_{0}+{k}_{e}a$, for some positive constants ${e}_{0}$ and ${k}_{e}$. Although it is likely that the prey energy $e\left(a\right)$ eventually saturates with age, predation does not allow reaching high ages in prey and therefore our linear approximation appears to be a good approximation. The actual number of offspring born to a predator individual is assumed Poisson-distributed, with mean $f$. Individual predators suffer from background mortality such that each predator dies with probability $m$ per time step.

Finally, we assume that the predator trait ${k}_{w}$ shaping the relationship between prey vitality and predator willingness to attack prey is traded off with another predator trait, so as to prevent runaway evolution to ${k}_{w}=0$, corresponding to the maximum predator attack rate on prey of any age and any mortality profile. Increasingly lower ${k}_{w}$ means that predators search for and attack increasingly more vital prey (Fig. 1c) and need thus invest more to maintenance as opposed to reproduction. We model this as an increase in the maintenance parameter $k$ in formula (6) with decreasing ${k}_{w}$, and let

7 $$k = \frac{1}{{1 + {\text{exp}}\left( {y k_{w} } \right)}}$$

for some positive trade-off strength $y$.

### Predator–prey eco-evolutionary dynamics

Within each time step, prey first mate and reproduce. We assume that mates are chosen randomly, regardless of their phenotypes. Upon mating, a Poisson-distributed number of offspring are produced, with mean $b(x,{k}_{d})$, where ${k}_{d}$ is the trait of a “mother” randomly chosen from the mating pair. The offspring are born with age 1 (as we emphasize earlier, fecundity already accounts for the first-timestep mortality). The phenotype ${k}_{d}$ of each prey offspring is then determined as follows. First, each offspring inherits the trait value from one of its parents, with equal probability. Then, with probability ${p}_{m}$ mutation occurs on this inherited trait. Upon mutation, a value generated from a normal distribution with zero mean and variance ${\sigma }_{m}^{2}$ is added to the offspring’s trait value (the trait value is set to zero if it would become negative).

Background mortality of other prey than the offspring then occurs: individual prey die each with their respective intrinsic mortality probability $d\left(a\right)$. This is followed by the extra mortality due to predation, described above and excluding just produced prey offspring. The age of all surviving prey individuals is then augmented by 1 and we record the trait distribution of the prey population (and calculate its mean and variance). Eventually, a maximum of $N$ prey are randomly selected to form the population at the beginning of the next time step.

Each predator then mates and reproduces. Also here, mates are chosen randomly, regardless of their phenotypes. Upon mating, a Poisson-distributed number of offspring are produced, with mean $f$. If evolution works also on predators, the phenotype ${k}_{w}$ of each predator offspring is determined analogously as in prey. Natural mortality of other predators than the offspring then follows. Finally, a maximum of $P$ predators are randomly selected to form the population at the beginning of the next time step.

We note that we also considered a more complex model of predator and prey genetics, assuming that both ${k}_{d}$ and ${k}_{w}$ were polygenic traits, represented by a large number of haploid loci with additive allelic effects among loci (a variant of the procedure due to Holt, Gomulkiewicz, and Barfield [38]). The results of the model involving this quantitative genetic step were analogous to those produced by the simpler evolutionary model described above.

### Pairwise invasibility plots

Pairwise invasibility plot (commonly abbreviated to PIP) is a standard way of visualizing evolutionary dynamics of a single trait, assuming that the timescale at which ecological dynamics operate is much faster than the timescale of evolution [39]. In its original form, it plots, for each pair of resident and mutant traits, fitness of a rare mutant entering a resident population at a stable ecological attractor (most commonly at a stable equilibrium). The idea is that when the resident and mutant traits are close to one another, the mutant either replaces the resident (if it has positive fitness) or fades away (when it has negative fitness). Evolution is then viewed as a sequence of small mutations followed by trait replacements and PIPs are a way to visualize and follow such mutation-replacement trait dynamics [39]. In particular, when mutations are small, evolution proceeds along the PIP diagonal, as any point on this diagonal stands for a single-trait population only. Invasion of mutants with a higher trait means moving in the PIP vertically slightly above the diagonal, and if such invasion is successful (trait replacement occurs) mutants become the new residents and we return to the PIP diagonal horizontally. If the invasion is not successful, we return to the PIP diagonal in the vertical direction, as nothing actually happened in the evolutionary sense. Analogously, invasion of mutants with a lower trait means moving in the PIP vertically slightly below the diagonal. In any case, another mutation is then assumed. This sequence of mutations and replacements stops either at an intermediate trait value or at a border of an admitted interval of the trait values.

The PIPs we present below are a stochastic variant of PIPs commonly presented in the literature, and we compose them in the following way. For each selected combination of resident and mutant traits, we first let the resident population settle at its ecological attractor via running its dynamics for 1000 time steps. Then, we add a small number of mutants and follow resident-mutant competition dynamics for other 2000 time steps. We then record the proportion of mutants in the final population. Very low proportions indicate that mutants fade away, whereas high proportions indicate that mutants eventually replace residents. The proportion of mutants in the final population thus in our case plays the role of fitness in the original PIP construction.

## Results

### Evolution of prey aging rate in absence of predators

**Fig. 2:**
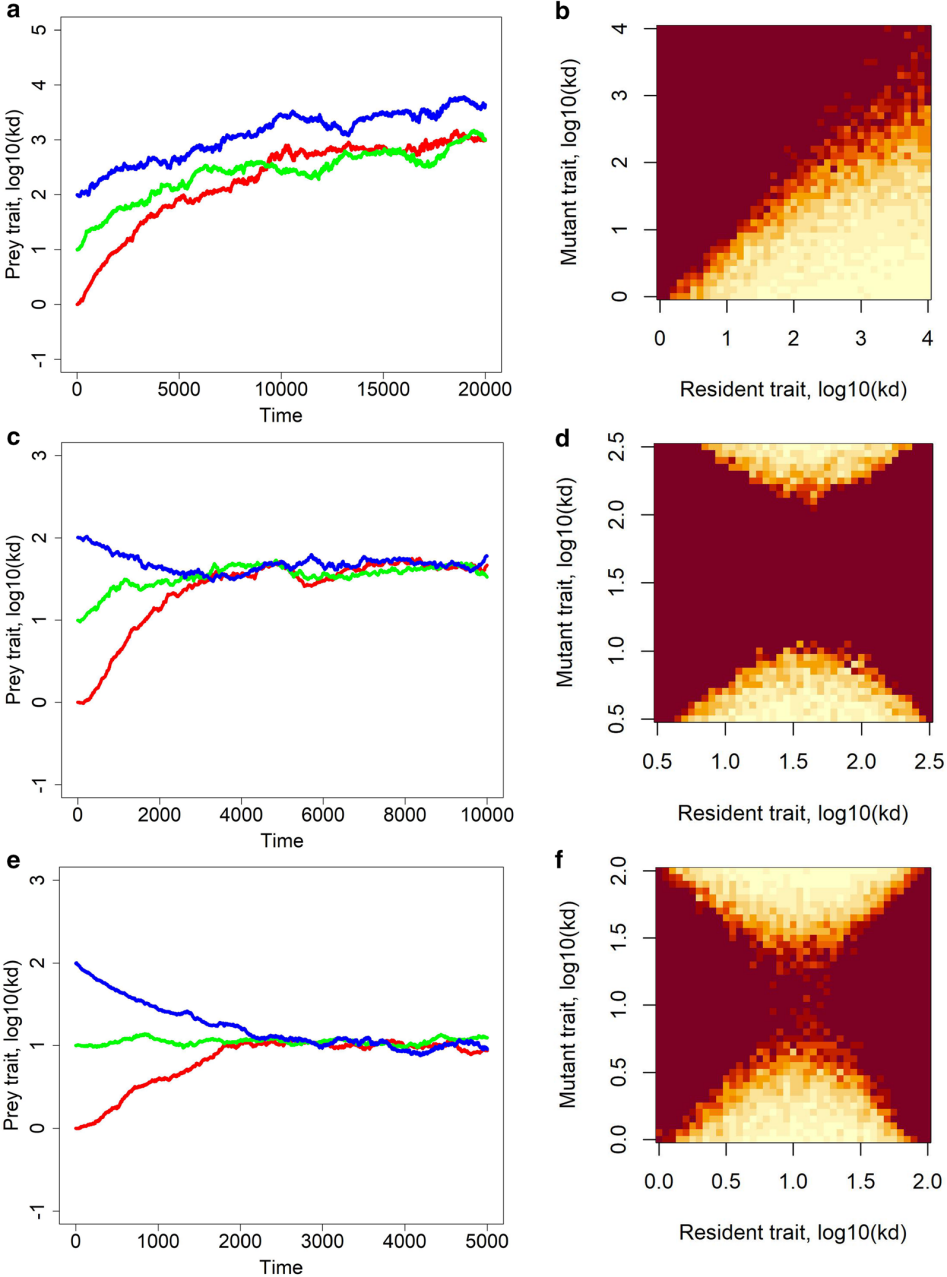
Evolution of the aging rate in prey ${k}_{d}$ in the absence of predators when there is no cost of aging ($x=0$, **a**, **b**), a very low cost ($x=0.001$, **c**, **d**) or a higher cost ($x=0.01$, **e**, **f**). The left panels (**a**, **c**, **e**) show one replicate of the temporal course of evolution for three different initial mean values of ${k}_{d}$; mean ${k}_{d}$ over the prey population is shown. Various replicates look very similar so we do not plot them here. The right panels (**b**, **d**, **f**) are the respective pairwise invasibility plots: while bright colors correspond to low proportions of mutants in the population and hence indicate mutant extinction, dark colors correspond to high proportions of mutants in the population and hence indicate that mutants would eventually replace residents. Other parameters are as in Table 1

We start with exploring the evolution of prey aging rate in the absence of predators. This represents the baseline scenario with which we compare our other results. When there is no cost of aging ($x=0$), prey age at an increasingly slower rate, as ${k}_{d}$ steadily increases, but there is no apparent low positive limit on the aging rate, that is, no upper limit on ${k}_{d}$ (Fig. 2, top row).

When a cost of aging is present, the prey trait ${k}_{d}$ appears to stabilize (Fig. 2, middle row). Hence, there appears to be an optimal aging rate modulated by the aging rate-fecundity trade-off. Indeed, ${k}_{d}$ attains lower values by evolution (that is, faster aging occurs) when the aging cost is higher (Fig. 2, bottom row).

### Evolution of prey aging rate in presence of predators

We now examine the evolution of prey aging rate in the presence of predators. The aging rates attained by evolution in the previous section are the optimal aging rates set by particular strengths of the aging rate-fecundity tradeoff, in the absence of predators. In line with the Williams’ hypothesis, we expect that with predators present prey cannot evolve slower aging rate than without predators. Rather, we expect that predator presence will lead to faster prey aging, and be shaped by predator characteristics.

**Fig. 3:**
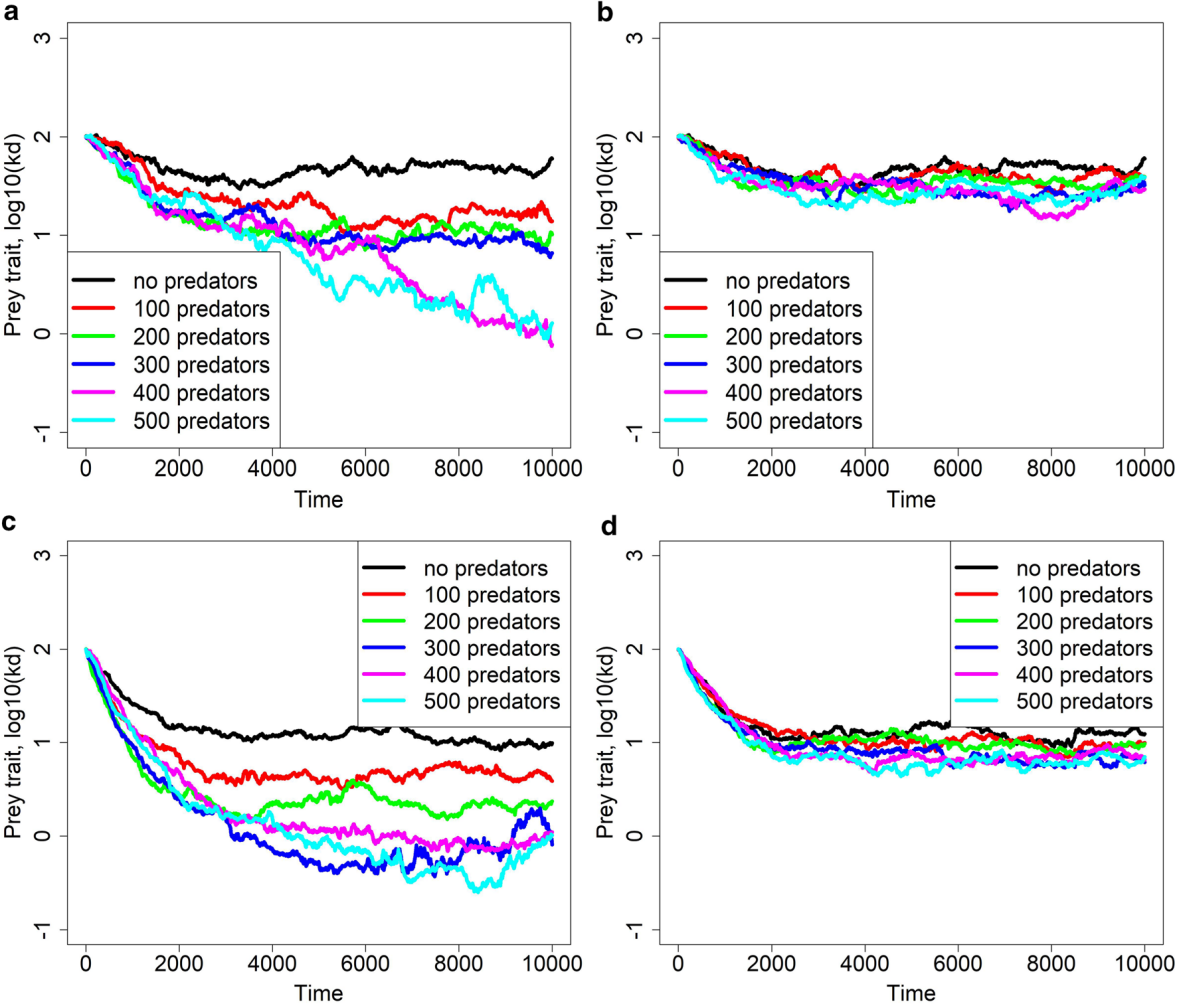
Evolution of the aging rate in prey ${k}_{d}$ in the presence of predators under several predator numbers, two (fixed) values of the predator trait ${k}_{w}$ (panels **a **and **c** are for ${k}_{w}=0.01$, **b** and **d** are for ${k}_{w}=100$), and two values of aging costs (**a** and **b** correspond to a very low cost of aging $x=0.001$ and **c** and **d** to a higher cost $x=0.01$). One typical replicate of temporal course of evolution for each value of ${k}_{w}$ is shown for clarity; the initial mean value of ${k}_{d}$ in prey is always set to 100. Other parameters are as in Table 1. Predators were not allowed to evolve and all have the same value of ${k}_{w}$. The no-predator scenario is shown in black

Figure 3 shows an effect of a different predator trait value ${k}_{w}$ and of a different number of predators. We recall that predators with lower ${k}_{w}$ have an increased diet breadth towards more vital (thus relatively younger) prey individuals. If ${k}_{w}$ is relatively large so that only relatively older individuals form predator diet, the optimal prey strategy does not differ too much from that without predators, which is to age relatively slowly close to the lower bound set by the aging rate-fecundity tradeoff. On the other hand, when predators consume also relatively young prey (such as when they have negative ${k}_{w}$), the optimal prey strategy appears to be to age faster and rather produce as many offspring as possible as soon as they can (so lower ${k}_{d}$ and in turn higher fecundity $b\left(x,{k}_{d}\right)$ evolves; Fig. 3). Moreover, while the number of predators does not appear to affect evolutionary endpoints when ${k}_{w}$ is relatively large, lower values of ${k}_{d}$ (i.e. faster aging) are attained if there are more predators around when ${k}_{w}$ is relatively small (Fig. 3). Hence, in the latter case, the Williams’ hypothesis appears to hold.

In Additional file 1: Fig. S1, we conduct a further sensitivity analysis and plot evolutionary trajectories of the prey aging rate ${k}_{d}$ under parameter values not considered here. We show there that while an increased energy needed to produce one predator offspring ${e}_{1}$ drives higher values of ${k}_{d}$ (i.e. slower aging rate), enhanced prey birth rate ${b}_{0}$ or minimum and maximum predator attack rates ${w}_{d0}$ and ${w}_{1}$, respectively, cause the evolved parameter ${k}_{d}$ to decrease (i.e. faster aging rate). In addition, the increased prey population $N$ or predator mortality $m$ do not have a pronounced effect for the corresponding adopted parameter value shifts.

### Coevolution

**Fig. 4:**
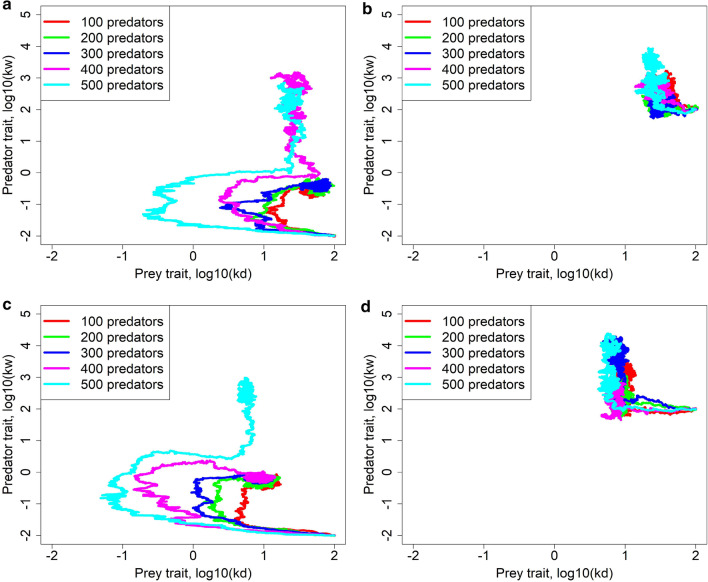
Coevolution of the prey trait ${k}_{d}$ and the predator trait ${k}_{w}$, for a very low cost of aging ($x=0.001$, **a**, **b**) or a higher cost ($x=0.01$, **c**, **d**). One replicate of temporal course of coevolution is shown for diverse numbers of predators, starting with two different values of ${k}_{w}$ (other replicates are quantitatively comparable). Other parameters are as in Table 1. The simulations were run for 40,000 time steps

Finally, we study coevolution between the prey trait ${k}_{d}$ and the predator trait ${k}_{w}$. Interestingly, evolutionary dynamics differ substantially for different starting values of the predator trait ${k}_{w}$ (Fig. 4). Consider first the left panels of this figure. Negative staring values of ${k}_{w}$ lead to evolution of low values of ${k}_{d}$, since prey need to produce as many offspring as possible as fast as possible and slower aging thus has no obvious advantage. We recall that the lower is ${k}_{w}$ the more predators add to their diet more vital (thus relatively younger) prey (see Fig. 1c). When ${k}_{d}$ becomes low and prey thus age faster, the vitality of most prey individuals also becomes low. As a consequence, it is no more advantageous for predators to have low ${k}_{w}$ which means high maintenance $k$. Selection thus prefers higher values of the parameter ${k}_{w}$ which therefore starts to rise. When ${k}_{w}$ increases, it becomes advantageous for prey to increase ${k}_{d}$ and so its vitality at any age; ${k}_{d}$ thus increases, eventually reaching a value close to value set by the aging rate-fecundity trade-off in prey in the absence of predators. Now consider the right panels of Fig. 4. Relatively large starting values of ${k}_{w}$ make the prey trait ${k}_{d}$ quickly attain a value driven by the aging rate-fecundity trade-off in prey and ${k}_{w}$ then attains an optimal value for that ${k}_{d}$. These results also hold true for other parameter values, as exemplified in Additional file 1: Fig. S2.

Interestingly, while the prey trait $${k}_{d}$$ seems to always reach an optimal value there is more than one possible final value of predator trait $${k}_{w}$$ (Fig. 4 and Additional file 1: Fig. S3). Predators can either strongly specialize on older, weak prey which are easy targets (high values of $${k}_{w})$$ or attack also relatively younger prey (low values of $${k}_{w})$$. Figure 4 shows that both strategies are stable when the predators are few in numbers in comparison to prey, but high $${k}_{w}$$ values are optimal when there are more predators. Accordingly, with a proper prey-to-predator ratio and starting conditions, both low and high $${k}_{w}$$ can evolve (Fig. S3), making $${k}_{w}$$ bistable.

### No aging rate-fecundity trade-off

Recently, some researchers have argued against aging rate-fecundity trade-offs as drivers of senescence and life span evolution [40]. To respect these views, we have also conducted simulation experiments with predators but without the aging rate-fecundity trade-off in prey (i.e. $x=0$).

**Fig. 5:**
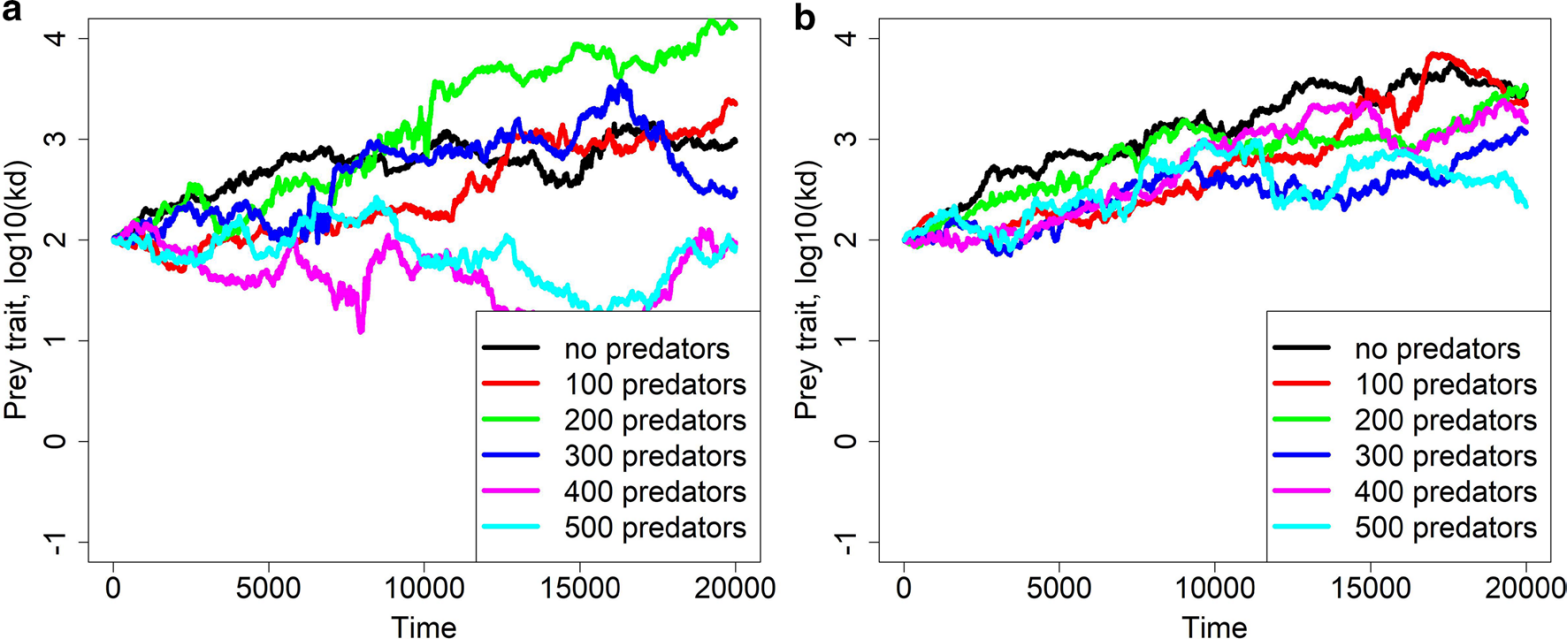
Evolution of the prey trait ${k}_{d}$ in the absence of aging rate-fecundity trade-off ($x=0$) yet in the presence of predators, under several numbers of predators and two (fixed) values of the predator trait ${k}_{w}$ (**a** is for ${k}_{w}=0.01$ and **b** for ${k}_{w}=100$). One replicate of temporal course of evolution for each value of ${k}_{w}$ is shown. Other parameters are as in Table 1. Predators were not allowed to evolve and all have the same value of ${k}_{w}$. The no-predator scenario is shown in black

**Fig. 6:**
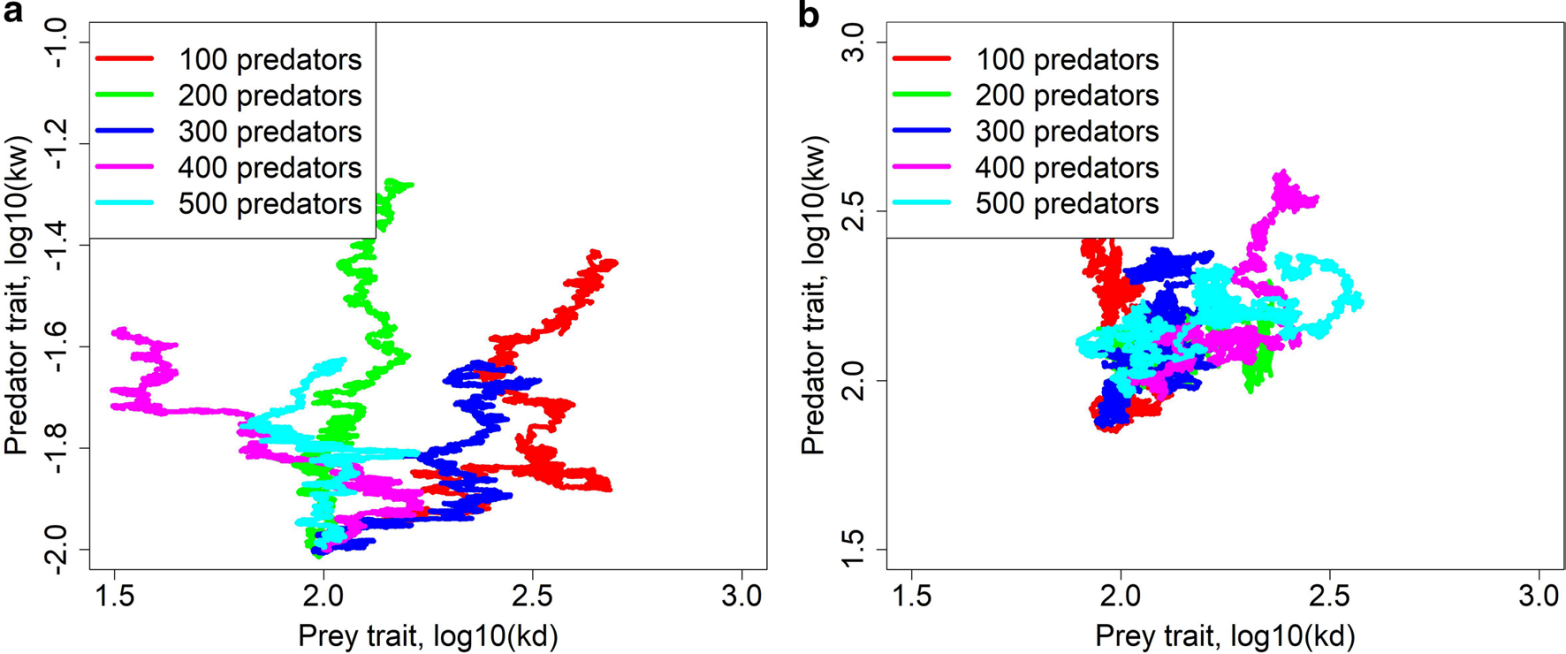
Coevolution of the prey trait ${k}_{d}$ and the predator trait ${k}_{w}$ in the absence of aging rate-fecundity trade-off ($x=0$). One replicate of temporal course of coevolution is shown for diverse numbers of predators, starting with two different values of ${k}_{w}$. Other parameters are as in Table 1. The simulations were run for 40,000 time steps

Interpretation of results, in this case, is not as clear as under the aging rate-fecundity trade-off in prey (Fig. 5). For example, some trajectories in Fig. 5 suggest that the presence of predators may speed up the evolution of slower aging rate in prey (${k}_{w}=-1$, left panel), but some contrarily suggest stabilizing selection (${k}_{w}=-2$, right panel). Such an ambiguity also remains with regards to the coevolution of prey and predator traits ${k}_{d}$ and ${k}_{w}$ (Fig. 6).

## Discussion

To summarize, our results show that if a trade-off between the pace of aging and fecundity exists, then this trade-off is the main factor setting the course of evolution of aging. Intense predation leads to somewhat faster aging. However, even then, the coevolution of predators and prey leads, in the long run, to the same pace of aging in prey as the evolution without predators. On the other hand, our simulations show that when the trade-off between aging and fecundity (in prey) is not present, the pace of aging evolves progressively slower without any lower limit. Furthermore, without the trade-off between aging and fecundity, predation becomes much more important and can lead to the evolution of both faster and slower aging, apparently at random. We hypothesize that this is because predation lowers the effective population size and thus, amplifies the effect of genetic drift.

Results of our coevolutionary simulations contradict a long-standing theoretical prediction that populations experiencing higher extrinsic mortality should always evolve to age faster [17, 41] as well as suggestions of some proponents of the programmed aging theory suggesting that higher extrinsic mortality should lead to slower aging [9, 42, 43]. More importantly, they may shed light on some previous experimental findings, e.g., that natural populations of *Daphnia ambigua* living in lakes varying dramatically in the intensity and duration of predation, age at the same pace [44]. Or that natural populations of guppies experiencing higher mortality do not evolve earlier set of senescence with regards to mortality or reproduction [30].

The model presented in this article is distinct from previously published models of the evolution of aging in two critical aspects. First and foremost, unlike most of the models published so far [42, 45–48], our model investigates the evolution of aging in organisms that reproduce sexually (i.e. mating is required) for reproduction rather than asexually. This is a critical point as, from a gene-centric view, an entire colony of asexual individuals is equivalent to a single individual, and thus it is hard to know if findings regarding the evolution of aging made on asexual models are translatable to sexually reproducing organisms. Second, our model focuses on the evolution of specific rates of aging instead of modeling competition between aging and non-aging individuals as some models do [46, 49]. In addition, our model is distinct simply because it focuses on a particular question that has not yet been investigated by previously published models. It shows that even this seemingly simple relationship between aging and external mortality is different and more complicated than evolutionary theoreticians anticipated.

At least three testable predictions may be derived from the results of our simulations. First, there is no trade-off between aging rate and fecundity in organisms with negligible senescence. In some organisms, this prediction may be challenging to test. However, in case of hydra, the absence of a trade-off between aging rate and fecundity seems to be almost certain. Hydra can live for hundreds or even thousands of years [2, 3], while its mortality and fertility are constant [3, 4]. If there was a trade-off between aging rate and fecundity in hydra it should reproduce particularly slowly if at all. This is, however, not the case. Thus, such trade-off in hydra most likely does not exist or is minuscule. Second, because the pace of aging varies considerably between species, the strength of trade-off between aging rate and fecundity must also vary considerably between different species and should be malleable by evolutionary forces as well.

Third, interventions aiming at the trade-off between aging rate and fecundity should be especially potent ways how to slow-down aging. This prediction is in a good agreement with a long-known fact that ablation of germline can, at least in some organisms, prolong life-span [50, 51]. However, our results suggest that the optimal approach to slow down aging should aim at modulating the pathways/signals regulating the trade-off between cellular maintenance and reproduction instead of focusing on one of its parts, i.e., germline.

## Conclusions

The results of this article show that the validity of William’s hypothesis depends on the presence of a trade-off between aging and fecundity and that it does not work if prey coevolves with predators. Overall, the results presented here provide a novel insight into the evolution of aging. They suggest an easy to grasp explanation of the great diversity of aging found in nature, including its absence, without a need to invoke controversial concepts such as programmed aging. Moreover, testable predictions based on these results translate into exciting opportunities for future research.

## Supplementary Information

**Additional file 1: **Sensitivity analysis.

## Data Availability

The datasets generated and/or analyzed during the current study available from the corresponding author on reasonable request.
