# Supplementary material for: Predation has small, short-term, and in certain conditions random effects on the evolution of aging
Source: BMC Ecol Evol. 2021 May 17;21:87. doi: 10.1186/s12862-021-01815-8 (PMC8130161; doi:10.1186/s12862-021-01815-8)
Supplement: Supplementary file 1 — Additional file 1: Sensitivity analysis. [file 12862_2021_1815_MOESM1_ESM.docx]

**Appendix**

**Sensitivity analysis**

In Fig. S1, we plot evolutionary trajectories of the prey aging rate $k_{d}$ under parameter values not considered in the main text, in order to examine sensitivity of the evolution across the parameter space. The black trajectory corresponds to the parameter set used in Fig. 3a in the main text for 200 predator individuals. Whereas an increased energy needed to produce one predator offspring $e_{1}$ drives higher values of $k_{d}$ (i.e. slower aging rate), enhanced prey birth rate $b_{0}$ or minimum and maximum predator attack rates $w_{d0}$ and $w_{1}$, respectively, cause the evolved parameter $k_{d}$ to decrease (i.e. faster aging rate). The increased prey population $N$ or predator mortality $m$ do not have a pronounced effect for the corresponding adopted parameter value shifts.


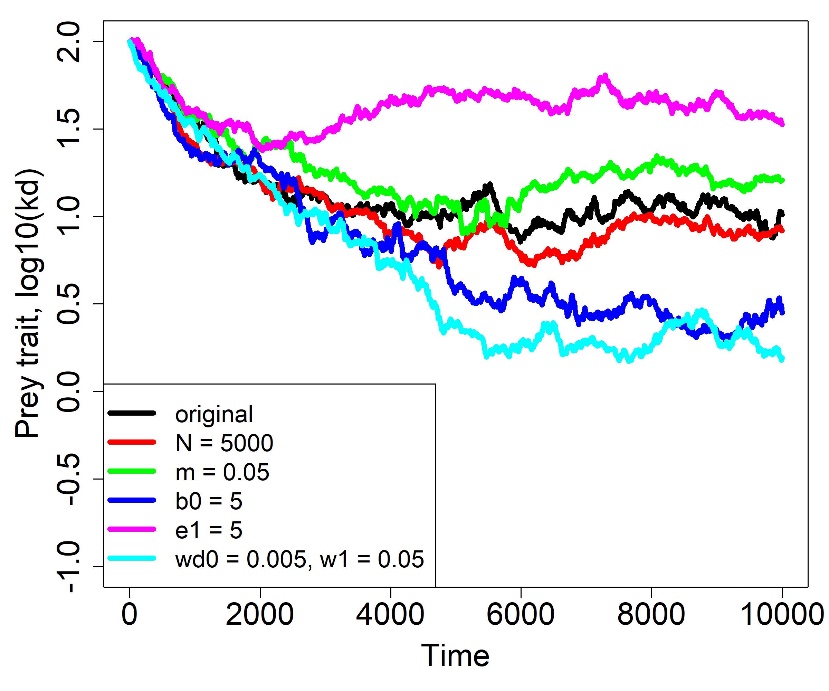


Figure S1: Evolution of the aging rate in prey $k_{d}$ in the presence of predators under several parameter sets. The baseline parameter set corresponds to that used in Fig. 3a in the main text for 200 predator individuals (original = black), with one parameter changed for each simulated trajectory; $x=0.001$. Predators were not allowed to evolve and all had the same value of $k_{w}$ = 0.01.

Coevolution of the prey and predator traits $k_{d}$ and $k_{w}$, respectively, also provides results analogous to those plotted in the main text for the baseline parameter set, as Fig. S2 shows.


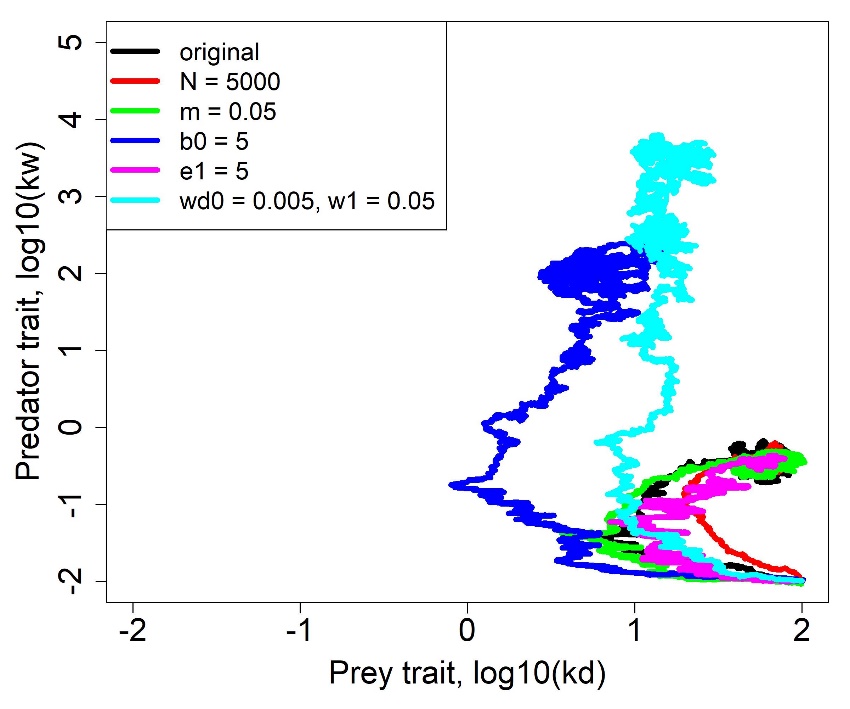


Figure S2: Coevolution of the prey trait $k_{d}$ and the predator trait $k_{w}$, for a very low cost of aging ($x=0.001$) and 200 predators. One replicate of temporal course of coevolution is shown for each parameter set (other replicates are quantitatively comparable). Other parameters are as in Table 1. The simulations were run for 40000 time steps.

**Evolutionary bistability**

As Fig. S3 clearly shows, coevolution between the prey aging rate trait $k_{d}$ and the predator attack rate trait $k_{w}$ can under some predator numbers lead to alternative stable evolutionary endpoints. Systems with relatively low numbers of predators end up either in the state with low $k_{w}$ (when starting at low $k_{w}$) or in the state with high $k_{w}$ (when starting at high $k_{w}$). Interestingly, however, the evolutionary stable value of $k_{d}$ is apparently similar in both cases and roughly corresponds to the value reached without predators (Fig. 2c in the main text). This is just an illustrative figure, but other parameter sets provide similar results.


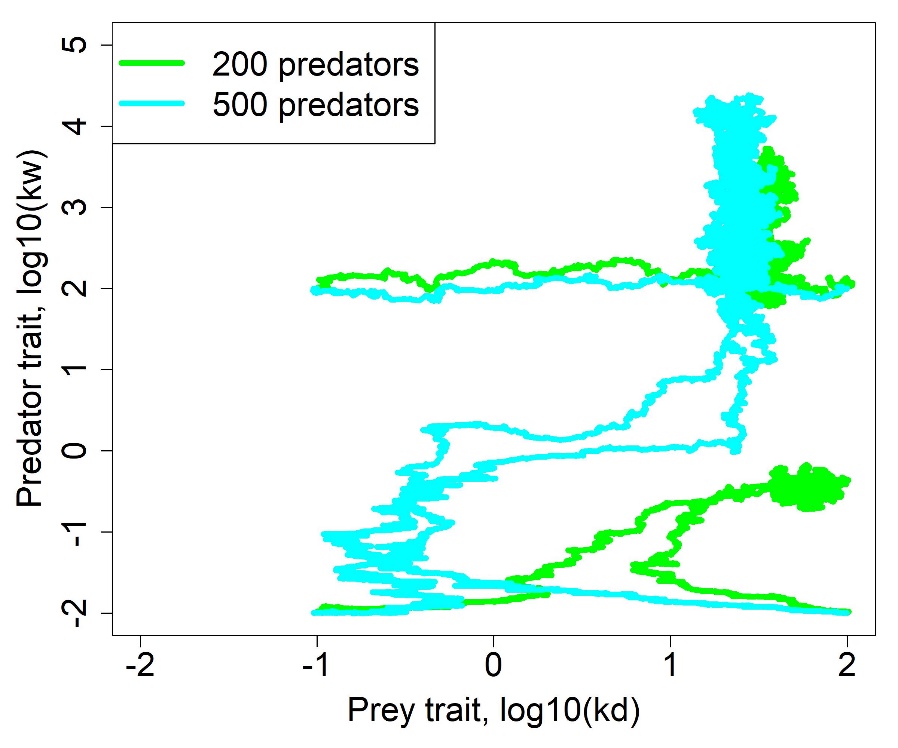


Figure S3: Coevolution between the prey aging rate trait $k_{d}$ and the predator attack rate trait $k_{w}$ can under two predator numbers. Four initial trait combinations are used in each case.

A possible interpretation of this evolutionary bistability is as follows. When prey are abundant relative to predators, then successful predators may have two alternative strategies: either focus on just the older prey where there is a greater chance to catch them (high $k_{w}$) or attempt to catch also relatively younger prey (low $k_{w}$). However, when the prey-to-predator ratio decreases (i.e. the number of predators per prey increases), the latter strategy is no more stable, since the predators that focus just on the older prey catch a lot of weaker prey, leaving those with lower $k_{w}$ frequently hungry as often needing to catch more vital prey. More generally, a strategy of predators adapted to an abundant prey population may become maladaptive when the predator population increases or the prey population declines.
